# Antitumor and cytotoxic activities of endophytic *Enterobacter hormaechei* derived secondary metabolites: *In-vitro* and *In-silico* study

**DOI:** 10.1371/journal.pone.0337344

**Published:** 2025-11-18

**Authors:** Muddasir Khan, Sumera Afzal Khan, Chieh-Wei Chang, Chien-Chin Chen, Bokyung Lee, Muhammad Hamayun

**Affiliations:** 1 Centre of Biotechnology and Microbiology, University of Peshawar, Peshawar, Pakistan; 2 Division of General Surgery, Department of Surgery, Ditmanson Medical Foundation Chia-Yi Christian Hospital, Chiayi, Taiwan; 3 Department of Pathology, Ditmanson Medical Foundation Chia-Yi Christian Hospital, Chiayi, Taiwan; 4 Department of Cosmetic Science, Chia Nan University of Pharmacy and Science, Tainan, Taiwan; 5 Doctoral Program in Translational Medicine, National Chung Hsing University, Taichung, Taiwan; 6 Department of Biotechnology and Bioindustry Sciences, College of Bioscience and Biotechnology, National Cheng Kung University, Tainan, Taiwan; 7 Department of Food Science and Nutrition, Dong-A University, Busan, South Korea; 8 Department of Health Sciences, the Graduate School of Dong-A University, Busan, South Korea; 9 Department of Botany, Abdul Wali Khan University Mardan, Mardan, Pakistan; University of Westminster - Regent Street Campus: University of Westminster, UNITED KINGDOM OF GREAT BRITAIN AND NORTHERN IRELAND

## Abstract

Anticancer therapies resistance, as well as their existing side effects, has become a significant issue worldwide. The demands of new anticancer agents that prevent cancer from developing and growing or spreading are increasing day by day. In this regard, our investigation assessed the cytotoxic and anticancer properties of secondary metabolites (SMs) obtained from understudied endophytic bacteria inhibiting *Alliaria petiolata*. The identified SMs were further screened by computational analysis against angiogenic factors of cancer. As a result, the leaf sample-associated isolate was identified as *Enterobacter hormaechei* AP2 strain. Gas chromatography-mass spectroscopy (GC-MS) analysis has shown the existence of 27 compounds present in the crude extract with main compounds being; 4,4-Ethylenedioxy-1-pentylamine (22.54%), Triethanolamine (15.17%) and 2-isobutoxyethyl acetate (12.51%). The extract showed anticancer activity with IC_50_ = 145 µM against the human glioblastoma cell line and cytotoxic activity with LC_50_ = 214.1 μg/mL against *Artemia salina* nauplii. The metabolite; 3-(2-Methylpropyl)hexahydropyrrolo[1,2-a]pyrazine-1,4-dione was predictively found most effective by computational analysis against angiogenic factors of cancer. It also demonstrated high intestinal solubility as well as low toxicity. In conclusion, the presence of *E. hormaechei* within *A. petiolata* may provide a wealth of bioactive chemicals. Validating the current discovery, purification, its biosynthesis route and other biological functions were recommended for additional research.

## Introduction

The notable global prevalence of cancer is due to the increasing risk factors that expose people to carcinogens. Normal cells are exposed to biological and chemical stressors by the risk factors that result in genetic alteration, proliferation and disruption of normal cell growth. Even though a variety of treatments are available to manage the condition but prevalence and mortality rates from cancer are still very high [1,2]. Chemotherapy, radiation and surgery are the primary pharmaceutical interventions for cancer. However, chemotherapy medications produce substantial organ damage which limits their usage at larger doses. Moreover, medication resistance has a significant impact on the efficacy of cancer treatments. New cancer medicines can be developed by focusing on elements implicated in cancer pathogenesis [3,4].

New medication therapy can be designed to prevent cancer from developing, growing or spreading. These medications can prevent angiogenesis, as the creation of blood vessels must sustain their growth and nourish cancer cells [5]. The two main angiogenic factors that cancer cells release are vascular endothelial growth factor (VEGF) and epidermal growth factor receptor (EGFR) [6,7]. These factors initiate endothelial cell proliferation, migration and invasion inside new vascular structures. Therefore, drugs that target these factors will limit the migration and proliferation of cancer cells and the subsequent attack on healthy cells. New anticancer drugs are constantly evolving in light of these factors [8].

In recent decades, bacteria have emerged as prolific producers of over two-thirds of natural bioactive compounds [9]. Notably, endophytic bacteria are symbionts, colonizing the tissues of their respective host plant. They offer diverse therapeutic potential for drug discovery, holding promise in combating disease [10,11]. They may be employed as bioactive agents and a source of natural chemicals to treat oxidative stress since they are connected to ethno-medicinal plants [12]. Although metabolites of these bacteria are not essential for their growth, they have an adaptive role in ecological interactions and environmental challenges by performing the role of a defensive compound or signaling molecule. They also generate low molecular weight secondary metabolites [13]. These compounds exhibit a wide range of medicinal presentations including anticancer, antioxidant, anti-inflammatory, antibacterial and immunosuppressive properties [14].

Moreover, endophytes encompassing plant-dwelling bacteria remain underexplored for their therapeutic potential, as they exist globally in practically every plant species [15]. A medicinal plant, *Alliaria petiolata* (common name: garlic mustard), belongs to the mustard family (*Brassicaceae*). It was first identified in 1860 in North America. It is mainly found in Europe, Asia, Africa and Eastern to Northern Pakistan. Due to their spicy taste, they are commonly used for cooking purposes. It contains a variety of bioactive substances that are highly effective in medicine. Due to the availability of limited data on their pharmacological activities, scientists are highly attracted [16]. The chemical composition and possible therapeutic advantages of *A. petiolata* has also been the subject of recent study. As a result, secondary metabolites such flavonoids, phenolic acids, and glucosinolates have been identified. Notably, the study found a number of chemicals that were previously unknown in this species, expanding our knowledge of the chemical richness of the plant [17].

As far as we know, no comprehensive and systematic studies have been reported exploring endophytic bacteria inhibiting *A. petiolata* and their pharmacological studies. In this context, the current study was prompted to explore *A. petiolata* residing endophytic bacteria and its secondary metabolites via GC-MS techniques and screened for *in vitro* antitumor and cytotoxic activities. Moreover, computational analysis was conducted to explore the best fit possible antitumor agent among these secondary metabolites as well as their pharmacokinetic properties. This study will promote the discovery of novel bioactive metabolites.

## Materials and methods

### Bacterial isolation

*A. petiolata* plants in zipper bags were collected from Shamozai, Swat, Pakistan (34°41’5.3052’‘ N, 72°7’40.7676’‘ E). The plant was identified by Botanist at Department of Botany, Abdul Wali Khan University Mardan (AWKUM), Mardan 23200, Pakistan, under voucher no. AWKUM-BOT-089/2022. After properly cleaning the leaf samples with tap water to get rid of any dust or debris, they were rinsed with distilled water. After that, subjected to further surface sterilization treatment and divided into 0.5–1.0 cm segments [18]. Four leaf segments were placed (in triplicate) in each nutrient agar plate, followed by incubation at 37°C for 24–48 hours. Initially, the obtained colonies were purified on the basis of colony morphology, Gram staining and biochemical characteristics. The bacteria were further identified by *16SrRNA* gene sequencing by Macrogen Inc., Korea. The obtained sequences were uploaded into NCBI nucleotide BLAST tool followed by phylogenetic analysis statistical neighbor joining bootstrap method using MEGA 11 software. Further, the sequences were submitted into NCBI GenBank database for obtaining accession numbers [19].

### Production, extraction, and identification of secondary metabolites (SMs)

The selected bacteria were cultivated [20] with minor modifications. Briefly, bacteria were inoculated in 250 mL of Luria Bertani (LB) broth medium and incubated at 200 rpm shaking for seven days at 37°C. Then, cell-free supernatant was collected using sterile Whattman filter paper. After adding ethyl acetate (Sigma-Aldrich) in a 1:1 (v/v) ratio, the liquid was briskly stirred for 5–10 minutes. It was split into two layers and the supernatant was collected. Then, ethyl acetate was evaporated using a rotary evaporator to produce crude extract. The SMs found in the crude extract were identified using the described GC-MS method [20]. Briefly, a Rt-Q-Bond capillary column (30 m × 0.25 mm × 8 μm) was used for the GC-MS analysis. The oven temperature was first adjusted at 40°C for three minutes and then increased at a rate of 5°C per minute to 220°C. The carrier gas was helium at a 10:1 split ratio and 1.0 mL/min. The MS system ran at an ion source temperature of 250°C and an electron impact ionization voltage of 70 eV while the injector temperature was 250°C. At a rate of 10 spectra per second, mass spectra were gathered in the 40–660 m/z range. Using ChromaTOF-HRT1 software compounds were identified by comparing their spectra and retention indices with the NIST-17 library.

### *In-vitro* bioactivities of crude SMs extract

#### Antitumor assay.

The crystal violet test is a rapid, easy and accurate way to assess the anticancer activity of cultured cells. It involves separating adherent cells on culture plates after they die and then using crystal violet dye staining to assess the adherence of the surviving cells [21]. Currently, an assay was carried out with a few minor adjustments in accordance with protocol [22] at Cancer Cell Culture & Precision Oncomedicine Lab, Institute of Basic Medical Sciences, Khyber Medical University, Peshawar 25100, Pakistan. To put it briefly, the human glioblastoma cell line (U87-MG) was used to assay for anticancer efficacy in crude SMs extract. A 96-well plate was filled with 10,000 cells, 10% FBS, 90% DMEM-F12 medium, and 1% antibiotics. The dish was then incubated for 24–48 hours at 37°C. Following seeding, several amounts of crude SMs extract (200, 100, 50, 25, 12.5, and 6.25 μM) were applied, along with cisplatin (Highnoon Laboratories Ltd.) as a positive control and an untreated negative control (0μM). To find the crude SMs extract’s maximum half inhibitory concentration (IC_50_), the plate was incubated for an additional 48 hours. Following treatment, 50 μL of a 4% paraformaldehyde solution was added to fix the cells and 50 μL of 0.1% crystal violet was added for staining. The plate was cleaned with tap water and allowed to dry completely after 20 minutes. After adding 200 μL of glacial acetic acid (Merck, Germany) to each well, the absorbance at 630 nm was measured. A 96-well plate was used for the triplicate test.

#### Cytotoxic assay.

The brine shrimp lethality assay is crucial for early cytotoxicity testing because it has the potential to kill laboratory-grown larvae (*Artemia salina* nauplii) [23]. With a few minor adjustments, crude SMs extract was tested for cytotoxicity as previously described [24]. Before treatment, *A. salina* nauplii were hatched in artificial sea water that contained 30 g of sea salt and 1 L of distilled water. They were then incubated for 24–48 hours at 27–30°C with strong aeration and constant lighting. Following hatching, each set of 15 *A. salina* nauplii were treated with 50–1000 μg/mL of crude SMs extract. They were then incubated for 24–48 hours at 27–30°C with vigorous aeration and constant lighting. The positive control was 5% potassium dichromate while the negative control was pure artificial sea water. The assay was run in triplicate and the LC_50_ value was determined using probit analysis. The result was then compared to Clarkson’s toxicity index to determine the toxicity level. The mortality rate (%) was computed using the following formula: number of dead nauplii/ Initial quantity of live nauplii (control) × 100.

#### Statistical analysis.

Data was analyzed using GraphPad Prism 10.4.1 software. Antitumor and cytotoxic activities were conducted in triplicate, and mean variation was analyzed using a t-test. A one-way ANOVA test was employed to evaluate significance (p < 0.05).

#### Computational analysis of SMs against angiogenic factors.

A computational technique known as molecular docking, which predicts the affinity of binding between the ligand and receptor protein [25], was utilized in the present study. GC-MS identified compounds in the crude SMs extract were selected as ligands. The angiogenic factors including VEGF and EGFR were chosen as target proteins. The known inhibitors of VEGF and EGFR were utilized as standards. The three dimensional (3D) structures of ligands and target proteins were retrieved from the PubChem web server and the Protein Data Bank web server. The target protein and ligand were prepared according to the method [26]. Briefly, the extra ligand and water molecules were removed from target protein and active site was determined using BIOVIA Discovery Studio 2021 software. The sdf files of ligands were converted into pdb files format by using the same software. Docking was carried out in accordance with the methodology described by Yusof et al. [27]. Initially, all ligands were docked against target protein by using multi-docking PyRx virtual screening software. The results were validated by repeated docking analysis using Autodock Vina tools. The protein-ligand complex was examined and visualized using Chimaera UCSF and BIOVIA Discovery Studio 2021 software [26]. High binding affinity and the best model compound present in the crude SMs extract were further screened for pharmacokinetics characteristics using the online tool http://biosig.unimelb.edu.au/pkcsm/prediction (accessed on 1 March 2025), followed by the standard protocol [28].

## Results

### *Enterobacter hormaechei* isolation and identification

**Fig 1:**
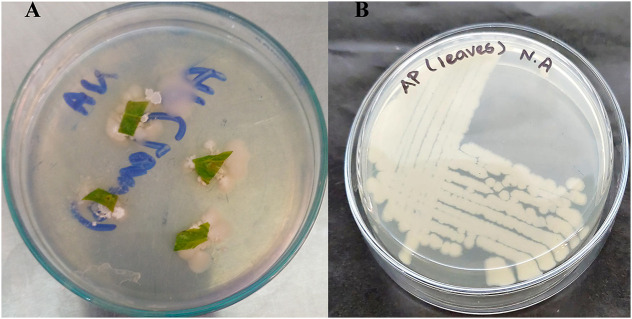
Endophytic *E. hormaechei* isolated from *A. petiolata*; A) After 24 hours of incubation, plant leaf samples display the initiation of endophytic bacteria on a nutrient agar plate and B) Isolate *E. hormaechei* growth on a nutrient agar plate.

**Fig 2:**
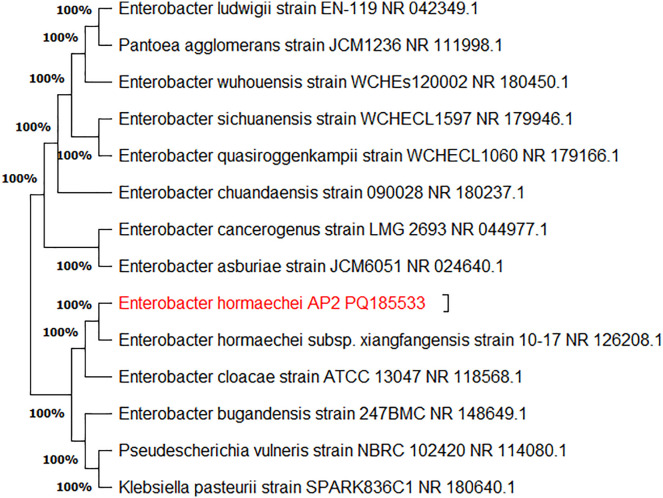
Phylogenetic tree of *E. hormaechei* strain AP2 constructed by MEGA 11 software following the statistical neighbor joining bootstrap method (Number of replications: 1000).

The collected fresh leaf samples were first cleaned of any dust or debris. Then, they were surface sterilized by immersing them in a 70% ethanol solution for five to ten minutes. Double-deionized distilled water was then used for washing. Following air drying, 0.5–1.0 cm pieces were placed on nutrient agar plates and incubated (Fig 1A). The purification of the bacterial isolate was achieved by repeatedly sub-culturing it on new nutrient agar plates. *Enterobacter hormaechei* (Fig 1B) was preliminarily identified as Gram-negative, rod-shaped, catalase positive, urease-positive, and was termed AP2. The obtained *16SrRNA* gene sequence was searched in the National Centre for Biotechnology Information (NCBI) for evolutionary relationships. The Nucleotide BLAST analysis showed the relationship of the AP2 isolate with the *E. hormaechei* strain. The sequence was submitted to NCBI GenBank under the accession number: PQ185533 (Fig 2).

### SMs production, extraction, and identification

*E. hormaechei* AP2 strain produced around 200 mg of crude SMs extract upon cultivation, using ethyl acetate (Sigma-Aldrich) as the solvent. GC-MS analysis of crude SMs extract showed the existence of 27 compounds with main compounds being; 4,4-Ethylenedioxy-1-pentylamine (22.54%), triethanolamine (15.17%) and 2-isobutoxyethyl acetate (12.51%), as listed in Table 1.

**Table 1:** The identified compounds by GC-MS analysis, present in the *E. hormaechei* strain AP2 crude SMs extract (S1 Fig).

Name	Retention Time	Relative retention index (RRI)	Area	Concentration (%)	Molecular formula	Molecular weight (g/mol)
2-(2-(2-Methoxyethoxy)ethoxy)ethyl acetate	6.274	627.4	5214361	4.44	C_9_H_18_O_5_	206.24
1,3,5-triazine-1,3,5(2*H*,4*H*,6*H*)-triethanol	8.340	834	504335	0.43	C_9_H_21_N_3_O_3_	219.28
2-Coumaranone	8.976	897.6	614481	0.52	C_8_H_6_O_2_	134.13
2,2-dimethyl-1,3-dioxolane	10.230	1023	5803798	4.94	C_6_H_12_O_3_	132.16
(Hydroxymethyl)ethylene acetate	11.695	1169.5	298317	0.25	C_7_H_12_O_5_	176.17
Triethanolamine	14.230	1423	17823918	15.17	C_6_H_15_NO_3_	149.19
2-isobutoxyethyl acetate	15.547	1554.7	14698109	12.51	C_8_H_16_O_3_	160.21
2,4-bis(1,1-dimethylethyl)phenol	15.779	1577.9	527343	0.45	C_14_H_22_O	206.32
Tributyl phosphate	16.020	1602	691313	0.59	C_12_H_27_O_4_P	266.31
Isopropyldiethanolamine	17.471	1747.1	3774312	3.21	C_7_H_17_NO_2_	147.22
Tetradecane	17.771	1777.1	869810	0.74	C_14_H_30_	198.39
Ethanol, 2,2′-[1,2-ethanediylbis(oxy)]bis-, diacetate	18.295	1829.5	6749168	5.74	C_10_H_18_O_6_	234.24
2,6,11-trimethyldodecane	18.800	1880	694192	0.59	C_15_H_32_	212.41
Carbamic acid, ((methylnitrosamino)methyl)-, isopropyl ester	19.423	1942.3	1981849	1.69	C_6_H_13_N_3_O_3_	175.19
2,6,11-trimethyldecane	20.162	2016.2	1032504	0.88	C_15_H_32_	212.41
3,3,4-trimethyldecane	20.478	2047.8	2899398	2.47	C_13_H_26_	182.35
Hexadecane	20.973	2097.3	1598989	1.36	C_16_H_34_	226.44
4,4-Ethylenedioxy-1-pentylamine	21.348	2134.8	26491956	22.54	C_7_H_15_NO_2_	145.20
3-methyl-dodecane	21.564	2156.4	1022375	0.87	C_13_H_28_	184.36
Hexahydro-Pyrrolo[1,2-a]pyrazine-1,4-dione	21.690	2169	3581484	3.05	C_7_H_10_N_2_O_2_	154.17
*N*-Decanoylmorpholine	23.270	2327	1432328	1.22	C_14_H_27_NO_2_	241.37
Pentadecanoic acid	23.595	2359.5	1459999	1.24	C_15_H_30_O_2_	242.40
2-methyl-2-(1-methylethyl)-1,3-dioxolane	23.994	2399.4	2418435	2.06	C_7_H_14_O_2_	178.65
3-(2-Methylpropyl)hexahydropyrrolo[1,2-a]pyrazine-1,4-dione	24.602	2460.2	3894248	3.31	C_11_H_18_N_2_O_2_	210.27
*n*-Hexadecanoic acid	25.682	2568.2	5940261	5.05	C_16_H_32_O_2_	256.42
Hexadecanoic acid ethyl ester	26.092	2609.2	1690594	1.44	C_18_H_36_O_2_	284.5
*Z*-11-hexadecenoic acid	27.263	2726.3	3819717	3.25	C_16_H_30_O_2_	254.41

### *In-vitro* bioactivities of crude SMs extract

#### Antitumor assay.

**Fig 3:**
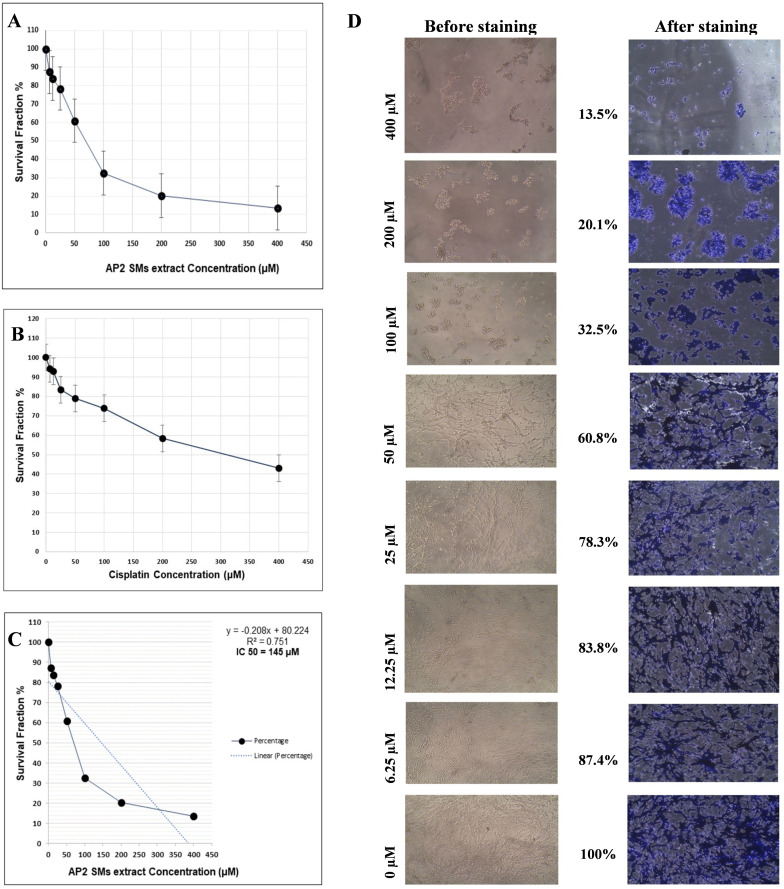
Antitumor activity of crude SMs extract; A and B) Survival fraction (%) of human glioblastoma cell line (U87-MG) against treatment of crude SMs extract and cisplatin (positive control), C) IC_50_ value graph of crude SMs extract treatment and D) After treatment microscopic images of human glioblastoma cell line, before and after crystal violet staining at different concentration, as at high concentration treatment it shows negotiable number of attached cells.

The crude SMs extract was tested against the human glioblastoma cell line (U87-MG) at doses ranging from 0 µM to 400 µM. With an IC_50_ of 145 µM against the human glioblastoma cell line, the extract has shown to be considerably effective (p < 0.05) lower then positive control (IC_50_ = 312 µM) (Fig 3A–3C). The cell shape was significantly impacted by higher quantities of SMs extract both before and after crystal violet stain, as shown in Fig 3D.

#### Cytotoxic assay.

**Fig 4:**
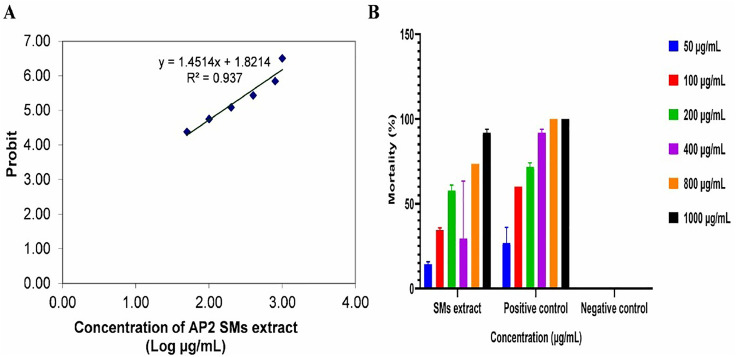
Cytotoxic activity of crude SMs extract; A) Probit vs. log concentrations of SMs extract graph and B) Significant (p < 0.05) percentage mortality of *A. salina* nauplii.

The cytotoxicity of crude SMs extract in concentrations ranging from 50 to 1000 μg/mL was checked by the brine shrimp lethality method against *A. salina* nauplii. The extract was found significant (p < 0.05), further based on plotting probit versus log concentrations of crude SMs extract graph, the LC_50_ was found to be 214.1 μg/mL (Fig 4A). In comparison with Clarkson’s toxicity index, crude SMs extract was found medium toxic. The positive control was found highly toxic with LC_50_ = 97.4 μg/mL. The detailed mortality rate was presented in Fig 4B.

### Computational analysis of identified SMs against angiogenic factors

**Fig 5:**
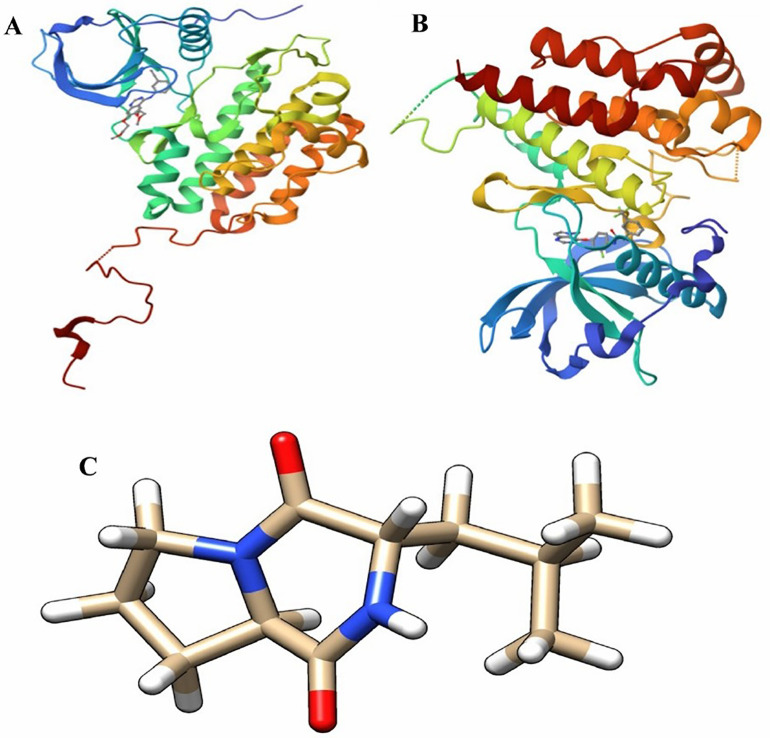
A) EGFR 3D structure with known kinase domain inhibitor gefitinib retrieved from protein data bank webserver (PDB ID: 1M17) and B) VEGF 3D structure with known kinase domain inhibitor sunitinib retrieved from protein data bank webserver (PDB ID: 3VHE) and C) 3-(2-Methylpropyl)hexahydropyrrolo[1,2-a]pyrazine-1,4-dione (PubChem CID: 7074739).

EGFR and VEGF, two angiogenic factors were chosen as target proteins. The protein data bank website provided the EGFR 3D structure with the known kinase domain inhibitor gefitinib (PDB ID: 1m17) (Fig 5A). On the other hand, the protein data bank website provided the VEGF 3D structure with the known kinase domain inhibitor sunitinib (PDB ID: 3vhe) (Fig 5B). Gefitinib and sunitinib were used as standard and for active site determination since they explicitly confirm the ATP binding site. The Pubchem website (S1 and S2 Tables) provided the 27 SMs’ 3D structures that were detected by GC-MS analysis. Using BIOVIA Discovery Studio 2021, the target proteins and ligands were made ready for molecular docking. Using multi-docking PyRx virtual screening software, all SMs were first docked to VEGF and EGFR proteins in order to identify the best SM with high binding affinities, which may be in charge of the extract’s anticancer action. Consequently, 3-(2-Methylpropyl)hexahydropyrrolo[1,2-a]pyrazine-1,4-dione (PubChem CID: 7074739) (Fig 5C) was named as **compound 1** due to its high binding affinity (−7.7) against EGFR and VEGF proteins, details binding affinities were presented in S1 and S2 Tables. Additionally, Autodock Tools 1.5.7 software was used to dock this compound once more in order to verify the outcome and examine its interaction.

**Fig 6:**
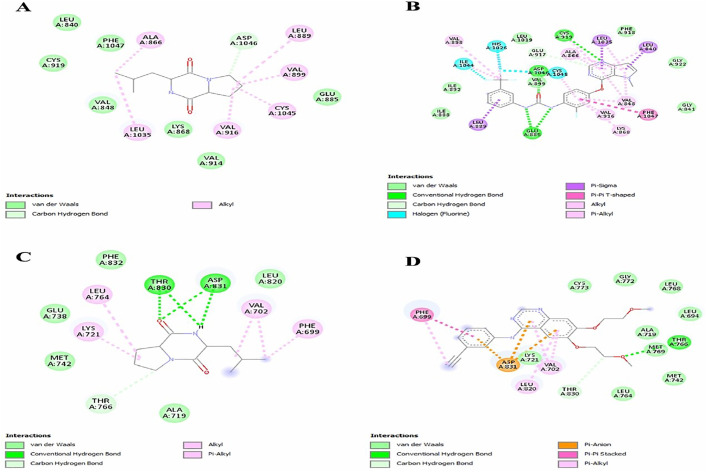
Protein-ligands complex, A) 3-(2-Methylpropyl)hexahydropyrrolo[1,2-a]pyrazine-1,4-dione docking complex with VEGF, B) Sunitinib docked as a standard, complex with VEGF, C) 3-(2-Methylpropyl)hexahydropyrrolo[1,2-a]pyrazine-1,4-dione docking complex with EGFR and D) Gefitinib docked as a standard, complex with EGFR.

In docking result with VEGF, **compound 1** and standard (sunitinib) demonstrated possible common alkyl bond with alanine (A: 866) and valine (A: 916) while **compound 1** has shown alkyl interaction with leucine (A: 1035) and with same residue standard shown pi-sigma interaction. **Compound 1** has shown alkyl interaction and standard shown halogen interaction with cysteine (A: 1045). **Compound 1** has shown alkyl interaction and standard shown van der waals interaction with valine (A: 899). **Compound 1** has shown alkyl interaction and standard shown pi-sigma interaction with leucine (A: 889) and with aspartic acid (A: 1046) **compound 1** displayed a carbon hydrogen bond whereas standard displayed a typical hydrogen bond; the whole interaction models were shown in Fig 6A and 6B.

In docking result with EGFR, **compound 1** and standard (gefitinib) demonstrated probable mutual van der waals interaction with alanine (A: 719) and alkyl bond with valine (A: 702) while with leucine (A: 820) **compound 1** has shown van der waals interaction and standard shown alkyl interaction. **Compound 1** has shown conventional hydrogen bond and standard shown carbon hydrogen bond with threonine (A: 830). C**ompound 1** has shown traditional bond of hydrogen and standard shown pi-anion bond with aspartic acid (A: 831). **Compound 1** has shown alkyl interaction and standard shown van der waals interaction with lysine (A: 721) and leucine (A: 764) while with phenylalanine (A: 699) **compound 1** has shown alkyl interaction and standard shown pi-pi stacked interaction, complete interaction models were presented in Fig 6C and 6D.

*In-silico* ADMET analysis was used to further evaluate **compound 1** for pharmacokinetic properties. With no hepatotoxicity, the findings showed intestinal absorption (84.241%), water solubility (−2.058 log mol/L), *T. pyriformis* toxicity (0.305 log ug/L) and minnow toxicity (2.465 log mM). The full ADMET attributes are compiled in Table 2.

**Table 2:** ADMET properties of 3-(2-Methylpropyl)hexahydropyrrolo[1,2-a]pyrazine-1,4-dione, secondary metabolites of *E. hormaechei* strain AP2.

Property	Model Name	Predicted Value
Adsorption	Water solubility	−2.058 log mol/L
	Caco2 permeability	1.186 log Papp in 10^−6^ cm/s
	Intestinal absorption (human)	84.241%
	Skin permeability	−3.971 log Kp
	P-glycoprotein substrate	No
	P-glycoprotein I inhibitor	No
	P-glycoprotein II inhibitor	No
Distribution	VDss (human)	0.104 log L/kg
	Fraction unbound (human)	0.6 Fu
	BBB permeability	−0.122 log BB
	CNS permeability	−2.992 log PS
Metabolism	CYP2D6 substrate	No
	CYP3A4 substrate	No
	CYP1A2 inhibitor	No
	CYP2C19 inhibitor	No
	CYP2C9 inhibitor	No
	CYP2D6 inhibitor	No
	CYP3A4 inhibitor	No
Excretion	Total clearance	1.214 log ml/min/kg
	Renal OCT2 substrate	No
Toxicity	AMES toxicity	No
	Max. tolerated dose (human)	0.618 log mg/kg/day
	hERG I inhibitor	No
	hERG II inhibitor	No
	Oral Rat Acute Toxicity (LD50)	2.027 mol/kg
	Oral rat chronic toxicity (LOAEL)	2.262 log mg/kg bw/day
	Hepatotoxicity	No
	Skin sensitization	No
	*T. pyriformis* toxicity	0.305 log ug/L
	Minnow toxicity	2.465 log mM

## Discussion

Despite the availability of a wide range of medicines, cancer is a leading cause of mortality worldwide. The resistance mechanisms of cancer cells significantly reduce the efficacy of existing cancer therapies. These therapies mainly concentrate on novel protein targets. Among these targets, VEGF and EGFR play a role in tumor cell proliferation and dissemination [1]. In bioactive microbial natural products, bacterial endophytes offer a rich source of bioactive secondary metabolites that can potentially treat cancer. They play a crucial role in the ongoing hunt for new cancer therapies that may target novel target proteins [1,29]. The computational study of the metabolites provides cost-effective and efficient methods for screening potential anticancer drugs [30].

This study was applied to identify and characterize bioactive metabolites of a bacterial endophyte isolated from the untapped *A. petiolata* leaf samples. This plant is widely found in moist to dry forest edges and used in traditional medicine as an antimicrobial, anti-inflammatory and for improving digestion [31]. However, no study reported their associated endophytes potential biological activities. So, for the first time *E. hormaechei* AP2 was isolated as an endophytic bacterium associated with *A. petiolata*, as they reported previously as an endophyte associated with *Persea americana* Mill. [32], *Stevia rebaudiana* [33], *Ananas comosus* L. [34], and *Allium sativum* L. [35] plants.

The endophytic bacteria produce numerous bioactive SMs including alkaloids, steroids, terpenoids, peptides, flavonoids, polyketones, quinols, and phenols [13]. Presently, GC-MS profile of *E. hormaechei* AP2 extracted SMs displays the existence of 27 bioactive metabolites. Among the *Enterobacter* specie, previously reported studies shown the existence of 2,4-bis(1,1-dimethylethyl)phenol [36], hexadecane [37], hexahydro-Pyrrolo[1,2-a]pyrazine-1,4-dione [38], 3-(2-Methylpropyl)hexahydropyrrolo[1,2-a]pyrazine-1,4-dione [39], *n*-Hexadecanoic acid [40], and hexadecanoic acid ethyl ester [41]. The remaining compounds were reported for the first time as secondary metabolites from *Enterobacter* specie including 2-(2-(2-Methoxyethoxy)ethoxy)ethyl acetate, 1,3,5-triazine-1,3,5(2*H*,4*H*,6*H*)-triethanol, 2-Coumaranone, 2,2-dimethyl-1,3-dioxolane, (Hydroxymethyl)ethylene acetate, triethanolamine, 2-isobutoxyethyl acetate, tributyl phosphate, isopropyldiethanolamine, tetradecane, ethanol, 2,2′-[1,2-ethanediylbis(oxy)]bis-, diacetate, 2,6,11-trimethyldodecane, carbamic acid, ((methylnitrosamino)methyl)-, isopropyl ester, 2,6,11-trimethyldecane, 3,3,4-trimethyldecane, 4,4-Ethylenedioxy-1-pentylamine, 3-methyl-dodecane, *N*-Decanoylmorpholine, pentadecanoic acid, 2-methyl-2-(1-methylethyl)-1,3-dioxolane, and *Z*-11-hexadecenoic acid.

The secondary metabolites identified in *E. hormaechei* AP2 derived crude extract, previously reported for various biological activities including 2-(2-(2-Methoxyethoxy)ethoxy)ethyl acetate reported for cytotoxic activity [42], 1,3,5-triazine-1,3,5(2*H*,4*H*,6*H*)-triethanol for acetylcholinesterase and butyrylcholinesterase inhibitory activities [43], 2-Coumaranone for nematicidal activity [44], 2,2-dimethyl-1,3-dioxolane for cytotoxic activity [45], (Hydroxymethyl)ethylene acetate for anti-ulcer activity [46], triethanolamine for antimicrobial activity [47], 2,4-bis(1,1-dimethylethyl)phenol for antifungal activity [48], tributyl phosphate for inhibition of neurogenesis [49], tetradecane for antimicrobial activity [50], ethanol, 2,2′-[1,2-ethanediylbis(oxy)]bis-, diacetate for textile application [51], 2,6,11-trimethyldodecane for antioxidant and anti-diabetic activities [52], 3,3,4-trimethyldecane for antimicrobial activity [53], 4,4-Ethylenedioxy-1-pentylamine for anti-diabetic and anti-inflammatory activities [29], hexahydro-Pyrrolo[1,2-a]pyrazine-1,4-dione for antioxidant activity [54], pentadecanoic acid for antifungal activity [55], 3-(2-Methylpropyl)hexahydropyrrolo[1,2-a]pyrazine-1,4-dione for antimicrobial activity [55], *n*-Hexadecanoic acid for antibacterial and antioxidant activity [56], hexadecanoic acid ethyl ester for antibacterial activity [57], and *Z*-11-hexadecenoic acid for antioxidant and cholinesterase inhibitory activities [58].

Bacterial endophytes-derived SM extract can combat various types of cancer cells [15,59,60]. Endophytic *E. hormaechei* has multiple activities like antioxidant [32], antibacterial [61] and plant growth-promoting activity [62] which have been reported till now. Still, based on the literature study, no study has reported the antitumor ability of endophytic *E. hormaechei*-derived SM extract; this work was the first to reveal its anticancer potential. Since the crude SMs extract from the *E. hormaechei* AP2 strain has shown considerable activity against human glioblastoma cell lines (IC_50_ = 145 µM). This was significantly (p < 0.05) lower than that of the positive control cisplatin (IC_50_ = 312 µM). This suggests that the extract outperformed the conventional medication in terms of anticancer activity. Compared to cisplatin, which acts through a single main mechanism, the crude extract exhibits higher cytotoxic effects due to the existence of many bioactive secondary metabolites that work in concert on a variety of molecular targets. The specific chemicals causing this activity have not yet been determined, though, as the current work is based on crude extract; more fractionation, purification and mechanistic research are required to confirm these results. By raising the concentration of SMs extract, they also have an impact on cell shape. Similarly, Huang et al. [63] and Romero-Arguelles et al. [64] both found strong anticancer activity of the crude SMs extract from endophytic bacteria.

Toxicity studies are critical in maintaining the safety of natural products [65]. The brine shrimp lethality test is one of the most frequent bioassays used to assess the toxicity of compounds as a screening tool for pharmacological activity. This test used brine shrimp to assess lethality by determining the LC_50_ value, the concentration required to kill 50% of brine shrimp nauplii. This approach is often used as preliminary screening because of its simplicity, cost-effectiveness, and rapid results [24]. In current investigation by the brine shrimp lethality method, the cytotoxicity of SMs extract was found significant (p < 0.05) and moderately toxic with LC_50_ = 214.1 μg/mL while positive control was found highly toxic with LC_50_ = 97.4 μg/mL. Moreover, in comparison with control the extract showed less dose-dependent cytotoxic activity. The complex composition of the secondary metabolites in the combination may be the reason why the crude extract did not clearly show dose-dependent cytotoxic action. Synergistic or antagonistic interactions between various chemicals, the instability of active constituents at increasing concentrations, or the likelihood that just a fraction of metabolites contribute to the observed activity might all be reasons for the lack of a linear response. Crude extracts frequently exhibit such non-dose-dependent effects; hence, further fractionation and purification are necessary to pinpoint the precise bioactive ingredients causing cytotoxicity. These finding aligns with previous studies [65–67].

A computational analysis was conducted to explore the antitumor potential of crude SMs extract derived from the *E. hormaechei* AP2 strain. Among the 27 identified metabolites, 3-(2-Methylpropyl)hexahydropyrrolo[1,2-a]pyrazine-1,4-dione was found highly efficient (Binding affinity = −7.7) against target angiogenic factors including VEGF and EGFR. This compound is a homodetic cyclic peptide, mainly produced by microorganisms [68]. Various biological activities were reported previously for this compound including anticancer [69,70], antimicrobial [71] and antibiofilm [68]. Furthermore, the ADMET characteristics of 3-(2-Methylpropyl)hexahydropyrrolo[1,2-a]pyrazine-1,4-dione were determined, as besides the efficient docking result, safety is an essential factor in drug discovery [1]. As a result, it displays high intestinal solubility as well as low toxicity, which strongly suggests the compound as an anticancer agent. Low toxicity and carrying to the target place are crucial characteristics of a medicine during development [72]. Moreover, the 3-(2-Methylpropyl)hexahydropyrrolo[1,2-a]pyrazine-1,4-dione has shown comparable binding interactions to the known inhibitor utilized as a reference, according to molecular docking studies. Our compound’s potential as a promising lead chemical is supported by the overlap in critical bonding contacts with the active site residues which implies that they may have a similar mode of action. This concordance between the test compound and the standard compound emphasizes the therapeutic importance of the discovered metabolite and further confirms the accuracy of the docking results.

## Conclusion

In the present study, *E. hormaechei* AP2 associated with *A. petiolata* produce 27 bioactive SMs. The crude extract of SMs was found effective for antitumor and cytotoxic activities. In computational analysis, 3-(2-Methylpropyl)hexahydropyrrolo[1,2-a]pyrazine-1,4-dione metabolite was found potent against angiogenic factors VEGF and EGFR with high intestinal solubility and low toxicity. Consequently, SMs generated from endophytic *E. hormaechei* may provide a wealth of resources for creating new bioactive compounds. The docking results presented here should be regarded as preliminary and predictive, serving primarily to guide future studies involving purification and definitive structural elucidation of 3-(2-Methylpropyl)hexahydropyrrolo[1,2-a]pyrazine-1,4-dione.

## Supporting information

S1 Fig GC-MS spectra of the *E. hormaechei* strain AP2 crude SMs extract. (PNG)

S1 Table Binding affinities of *E. hormaechei* strain AP2 derived secondary metabolites against epidermal growth factor receptor (EGFR). (DOCX)

S2 Table Binding affinities of *E. hormaechei* strain AP2 derived secondary metabolites against vascular endothelial growth factor (VEGF). (DOCX)
