# Supplementary figures and images for: Antitumor and cytotoxic activities of endophytic Enterobacter hormaechei derived secondary metabolites: In-vitro and In-silico study
Source: PLoS One. 2025 Nov 18;20(11):e0337344. doi: 10.1371/journal.pone.0337344 (PMC12626318; doi:10.1371/journal.pone.0337344)

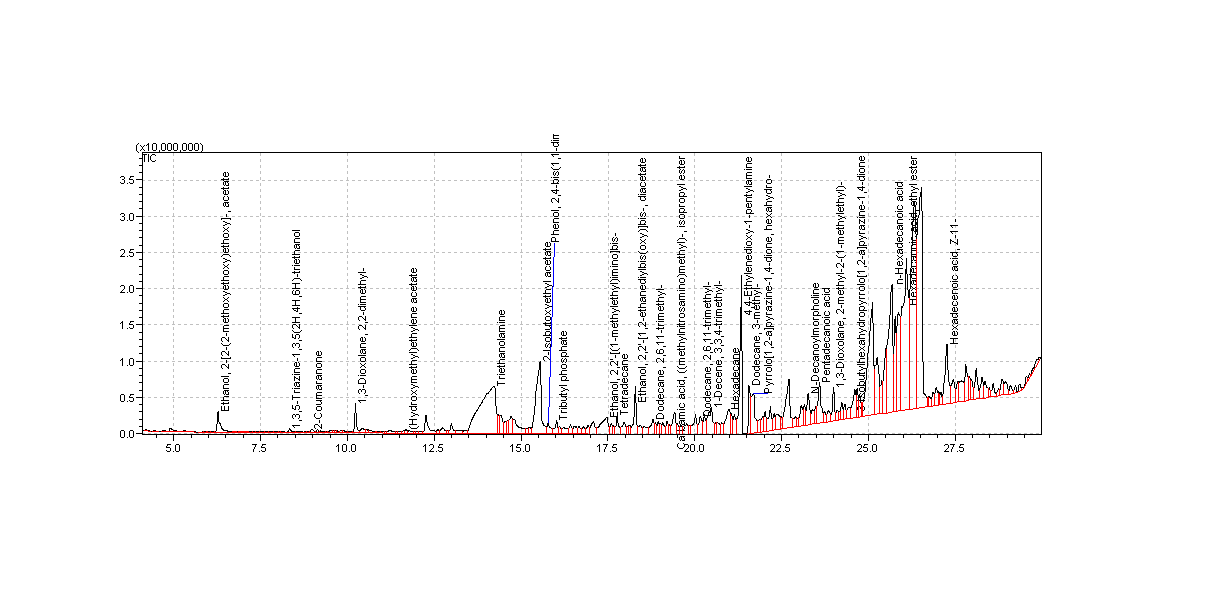

Supplement: S1 Fig — (PNG) [file pone.0337344.s001.PNG]
