# Supplementary material for: Antitumor and cytotoxic activities of endophytic Enterobacter hormaechei derived secondary metabolites: In-vitro and In-silico study
Source: PLoS One. 2025 Nov 18;20(11):e0337344. doi: 10.1371/journal.pone.0337344 (PMC12626318; doi:10.1371/journal.pone.0337344)
Supplement: S1 Table — (DOCX) [file pone.0337344.s002.docx]

#### S1 Table. Binding affinities of *E. hormaechei* strain AP2 derived secondary metabolites against epidermal growth factor receptor (EGFR)

| **PubChem CID#** | **Secondary metabolites** | **Binding affinities** |
| --- | --- | --- |
| 19218 | 2-(2-(2-Methoxyethoxy)ethoxy)ethyl acetate | -4.4 |
| 104556 | 1,3,5-triazine-1,3,5(2*H*,4*H*,6*H*)-triethanol | -5.4 |
| 68382 | 2-Coumaranone | -5.5 |
| 76209 | 2,2-dimethyl-1,3-dioxolane | -4.9 |
| 66021 | (Hydroxymethyl)ethylene acetate | -5.1 |
| 7618 | Triethanolamine | -4.5 |
| 520944 | 2-isobutoxyethyl acetate | -4.8 |
| 7311 | 2,4-bis(1,1-dimethylethyl)phenol | -7.0 |
| 31357 | Tributyl phosphate | -5.6 |
| 8498 | Isopropyldiethanolamine | -4.6 |
| 12389 | Tetradecane | -4.9 |
| 8098 | Ethanol, 2,2′-[1,2-ethanediylbis(oxy)]bis-, diacetate | -5.0 |
| 35768 | 2,6,11-trimethyldodecane | -5.5 |
| 51679 | Carbamic acid, ((methylnitrosamino)methyl)-, isopropyl ester | -5.4 |
| 35768 | 2,6,11-trimethyldecane | -5.6 |
| 39499 | 3,3,4-trimethyldecane | -5.1 |
| 11006 | Hexadecane | -5.1 |
| 560007 | 4,4-Ethylenedioxy-1-pentylamine | -4.7 |
| 56619966 | 3-methyl-dodecane | -4.9 |
| 11441559 | Hexahydro-Pyrrolo[1,2-a]pyrazine-1,4-dione | -6.6 |
| 94801 | *N*-Decanoylmorpholine | -5.7 |
| 13849 | Pentadecanoic acid | -5.3 |
| 12741631 | 2-methyl-2-(1-methylethyl)-1,3-dioxolane | -4.8 |
| 7074739 | 3-(2-Methylpropyl)hexahydropyrrolo[1,2-a]pyrazine-1,4-dione | -7.7 |
| 985 | *n*-Hexadecanoic acid | -5.3 |
| 12366 | Hexadecanoic acid ethyl ester | -5.1 |
| 5312414 | *Z*-11-hexadecenoic acid | -5.5 |
